# Analysis of 4000 kidney transplantations in a single center: Across immunological barriers

**DOI:** 10.1097/MD.0000000000004249

**Published:** 2016-08-12

**Authors:** Hyunwook Kwon, Young Hoon Kim, Ji Yoon Choi, Shin Sung, Joo Hee Jung, Su-Kil Park, Duck Jong Han

**Affiliations:** a Department of Surgery, Hallym University Sacred Heart Hospital, Hallym University College of Medicine, Pyeongchon Anyang; b Department of Surgery; c Division of Nephrology, Department of Internal Medicine, Asan Medical Center, University of Ulsan College of Medicine, Seoul, Korea.

**Keywords:** ABO-incompatible, desensitization, flow-cytometric crossmatch, kidney transplantation

## Abstract

Kidney transplant (KT) is the optimal renal replacement therapy for patients with end-stage renal disease (ESRD). The demand for kidneys, however, continues to exceed the supply. To overcome this problem, efforts to extend the donor pool by including human leukocyte antigen (HLA)- and ABO-incompatible (ABOi) KTs are increasing. The aim of this article was to retrospectively review data on recipients, donor profiles, and clinical outcomes in 4000 cases of KT. In addition, we analyzed clinical outcomes in ABOi and flow-cytometric crossmatch (FCXM) positive KT in a subgroup analysis.

This was a retrospective, observational study using data extracted from medical records. A total of 4000 consecutive patients who underwent KT at our institution from January 1990 to February 2015 were included in this study. KTs across immunological barriers such as ABO incompatible (276 cases, 6.9%), FCXM positive (97 cases, 2.4%), and positive complement-dependent cytotoxicity (CDC) XM KT (16 cases, 0.4%) were included.

From a Kaplan–Meier analysis, overall patient survival (PS) rates after KT at 1, 5, 10, and 20 years were 96.9%, 95.1%, 92.0%, and 88.9%, respectively. The overall graft survival (GS) rates after KT at 1, 5, 10, and 20 years were 96.3%, 88.9%, 81.2%, and 67.4%, respectively. Our subgroup analysis suggested that overall PS, GS, death-censored GS, and rejection-free GS in ABOi KT showed no significant differences in comparison with ABO-compatible KT if adequate immunosuppressive treatment was performed. The overall PS rate in patients who underwent FCXM positive KT did not differ significantly from that of the control group during the 3-year follow-up (*P* = 0.34). The overall GS, death-censored GS, and rejection-free GS also did not differ significantly between the FCXM KT and control groups (*P* = 0.99, 0.42, and 88).

The outcomes of KTs continually improved during the study period, while the annual number of KTs increased. ABO or FCXM positive KTs can be performed safely with successful graft outcomes.

## 1 Introduction

Kidney transplant (KT) is the optimal renal replacement therapy for patients with end-stage renal disease (ESRD) as it results in better quality of life and prolonged survival compared to hemodialysis or peritoneal dialysis. ^[1]^ The demand for kidneys, however, continues to exceed the supply. According to the Korean Network for Organ Sharing Annual Report 2012, 12,300 patients are still waiting for deceased-donor KTs (DDKTs) in Korea, despite the increase in the number of KTs in past decades (1783 KTs were performed in 2012). ^[2]^

To overcome this problem, there has been a worldwide evolution in KT in the past century largely attributed to advances in surgical techniques and knowledge and the expansion of donor sources. ^[3]^ In particular, efforts to extend the donor pool – including the use of expanded criteria donor grafts, complement-dependent cytotoxicity (CDC) positive crossmatch (XM) KTs, and ABO-incompatible (ABOi) KTs – have recently increased. ^[4]^ After the 1st KT at the Asan Medical Center (AMC) in 1990, the number of living and deceased KTs in our center has continuously increased through the utilization of multiorgan transplantation with KT and KT across ABO and anti-human leukocyte antigen (HLA) antibody (Ab), reaching 4000 in February 2015.

The aim of this article was to retrospectively review data on recipients, donor profiles, and clinical outcomes of KT. More specifically, we analyzed clinical outcomes in ABOi and flow-cytometric crossmatch (FCXM) positive KT.

## 2 Materials and methods

### 2.1 Patients and definitions

This was a retrospective, observational study using data extracted from medical records. A total of 4000 consecutive patients who underwent KT at AMC from January 1990 to February 2015 were included in the study. This study was approved by the Institutional Review Board of the AMC (2014-0776). Graft survival (GS) was defined as the time from transplantation to graft loss, return to dialysis, or last follow-up with a functioning graft. Data were censored for patient death with a functioning graft to analyze death censored GS. Rejection free GS was defined as the time from transplantation to biopsy-proven acute rejection.

### 2.2 Immunosuppression

**Figure 1:**
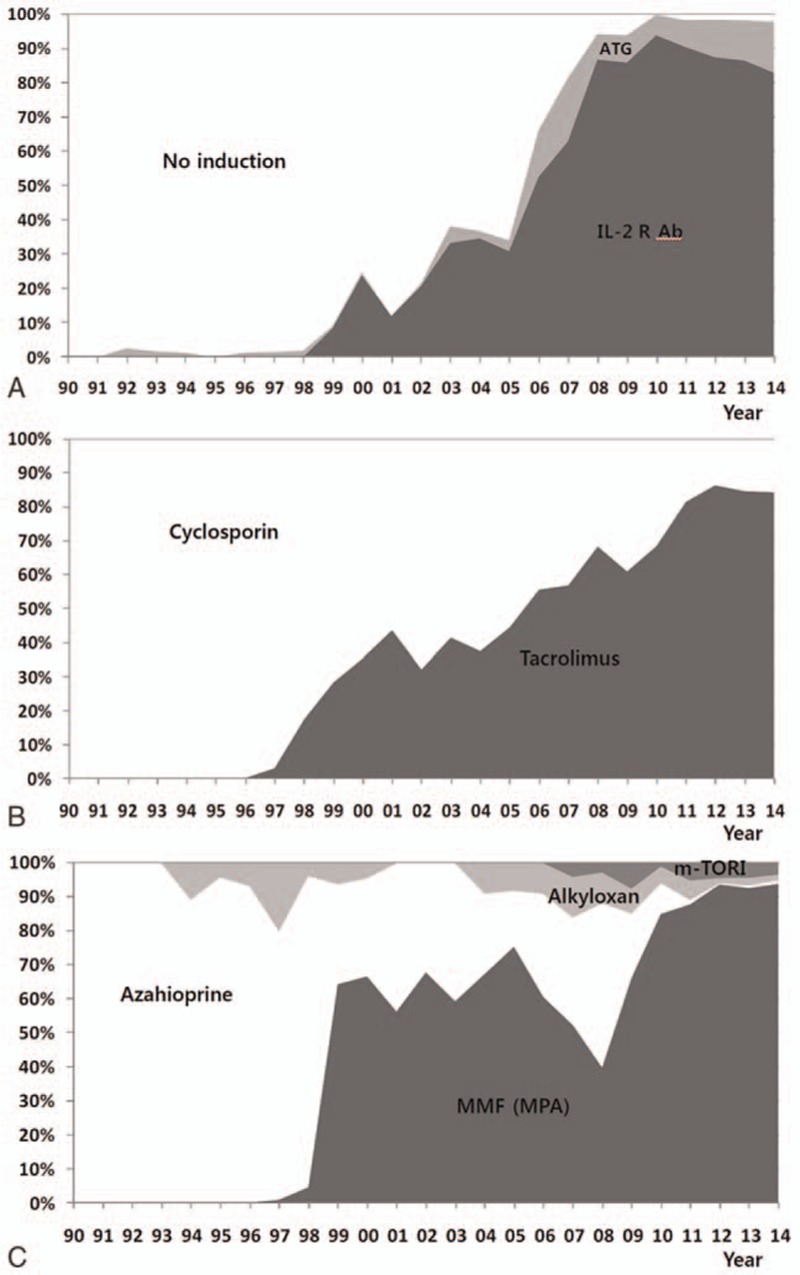
Immunosuppressive regimen during 4000 kidney transplantation (A) induction agent, (B) calcineurin inhibitor, and (C) antimetabolite. ATG = anti-thymocyte globulin, IL-2 R Ab = interleukin-2 receptor antibody, m-TORI = m-TOR inhibitor, MMF = mycophenolate mofetil, MPA = mycophenolic acid.

Initially, the immunosuppressive regimen consisted of a calcineurin inhibitor (CNI), azathioprine, and a corticosteroid without an induction regimen. However, the immunosuppressive regimen changed during the study period. Currently, the regimen includes a CNI (tacrolimus or cyclosporine), mycophenolic acid, and a corticosteroid. Alternatively, in the case of patients with immunological risk factors, such as highly sensitized individuals or those with previous graft loss due to rejection, and those who wish to avoid the long-term use of steroids, an induction regimen of rabbit antithymocyte globulin (Thymoglobulin; Genzyme, Cambridge, MA) is administered. The changes in the immunosuppressive regimen are shown in Fig. 1.

### 2.3 Subgroup analysis: ABO-incompatible and FCXM positive KT

To evaluate the clinical outcomes of ABOi KT and FCXM positive KT, a subgroup analysis was performed. Between January 2009 and February 2015 at the AMC, ABOi KT was performed on 276 patients and a total 96 patients underwent FCXM positive KT. A total of 42 patients who underwent ABOi and FCXM positive KT were excluded. The remaining 234 patients in the ABOi KT group were divided into era 1 (2009–2012) and era 2 (2012–2015) by desensitization protocol. Among the 54 patients in FCXM positive KT group, 9 patients who underwent CDC positive KT were excluded. The remaining 45 patients who underwent FCXM positive KT were included in the subgroup analysis. A total of 600 patients who underwent ABO compatible and FCXM negative KT from January 2012 to February 2015 served as a control group.

The standard tube method was used to determine ABO isoagglutinin titers. Type A or B red blood cell suspensions were incubated in 0.1 mL of saline containing 2-fold serial dilutions of the patient's serum at room temperature. The final dilution, with trace reactivity, was defined as the anti-A/B Ab titer in each assay. ABOi KT was performed under the condition that the IgG and IgM Ab titers against blood group A or B were below 1:8. Two-color FCXM analysis was performed using a BD FACS Canto II (BD Biosciences, San Jose, CA) instrument with the use of antihuman CD3-PerCP (peridinin chlorophyll protein, BD Biosciences) and antihuman IgG F[ab]′ fluorescein isothiocyanate (Jackson ImmunoResearch Laboratories, Inc., West Grove, PA). T-cell and B-cell FCXM were considered positive when the ratio of XM median values to the control median value (median fluorescence intensity ratio) exceeded 2.0 and 2.5, respectively. Conversion to a negative FCXM was the target of pretransplant desensitization. In our initial desensitization protocol (era 1), patients received a single dose of rituximab (500 mg) 1 week before plasmapheresis. We maintained the tacrolimus trough level at 8 to 10 ng/mL and administered 1.5 g/day of mycophenolate mofetil (MMF) during the 1st 3 months. After we experienced lethal infectious complications, a modified immunosuppression protocol was applied from January 2012 (era 2). We reduced the dose of rituximab from 500 to 200 mg in ABOi patients unless patients showed positive FCXM. Tacrolimus was given at an initial level of 8 ng/mL and reduced to 3 to 8 ng/mL 1 week after transplantation. The dose of MMF was reduced from 1.5 to 1 g/day after the 7th postoperative day. All ABOi KT patients received induction therapy with basiliximab (an anti-CD25 monoclonal Ab) on the day of KT and 4 days after surgery. We selectively used cyclosporine as a 1st line CNI for patients 55 years old or older. Those patients were also given acyclovir for CMV prophylaxis. Numbers of plasmapheresis and additional treatment, such as intravenous immune globulin or bortezomib, were dependent on the follow-up results of ABO Ab titers and FCXM results during desensitization treatment.

### 2.4 Statistical analysis

Categorical variables were presented as counts and percentages and analyzed using the Chi-square test and Fisher exact test, as appropriate. Continuous variables were presented as means ± standard deviations (SDs) and compared using Student *t* test and a one-way analysis of variance (ANOVA) as appropriate. Variables that were not distributed normally were analyzed using the Mann–Whitney *U* test and Kruskal–Wallis test. Patient survival (PS) and GS rates were calculated using the Kaplan–Meier method and compared using the log-rank test. All statistical analyses were carried out with SPSS software (version 18.0; SPSS, Chicago, IL), and *P* ≤ 0.05 was considered statistically significant.

## 3 Results

AMC performed the 1st KT with a living donor in 1990. In 1992, the 1st cadaveric simultaneous pancreas-kidney transplantation was performed in Korea. Since 1996, we have performed more than 100 KTs each year at our hospital. The indications for KT have expanded to a simultaneous multiorgan transplantation with positive CDC XM KT, and ABOi KT. In terms of multiorgan transplants, a simultaneous liver–KT was performed in 1999, a simultaneous heart–KT was performed in 2005, a living-donor simultaneous pancreas-kidney transplantation was performed in 2006, and a simultaneous cadaveric pancreas and living kidney transplant was performed in 2009, marking major milestone events in Korea. Our center has performed more than 300 KTs a year since 2012. From 1990, approximately 130 months were needed for the 1st 1000 KTs, while that figure was 84 months for the 2nd 1000, 57 months for the 3rd 1000, and 26 months for the most recent 1000. A total of 4000 KTs performed in our center between 1990 and 2015 were included in this study.

### 3.1 Patient demographic and clinical characteristics

Of the 4000 patients who received KTs at our hospital, 55.9% were male. The mean age was 41.3 ± 12.1 years (range, 17–73 years). The most common cause of ESRD was diabetes mellitus (16.1%), followed by glomerulonephritis (14.5%) and hypertension (9.3%). Approximately 13.8% of patients received preemptive KTs before renal replacement therapy. The median HLA and DR mismatches were 3 (interquartile range: 1.5–4.5) and 1 (interquartile range: 0.5–1.5), respectively. Out of 3021 patients, 435 (14.4%) were anti-HLA panel-reactive antibody (PRA) ≥20% before transplantation (class I, 13.1%; class II, 16.4%). There were 276 (6.9%) cases of ABOi, and 97 (2.4%) cases of FCXM-positive KTs, and 16 (0.4%) cases of CDC positive KTs. Of the 4000 donors, 3095 cases (77.4%) were living, and 905 cases (22.6%) were deceased. Pretransplant recipient and donor characteristics are listed in Tables 1 and 2, respectively.

**Table 1:**
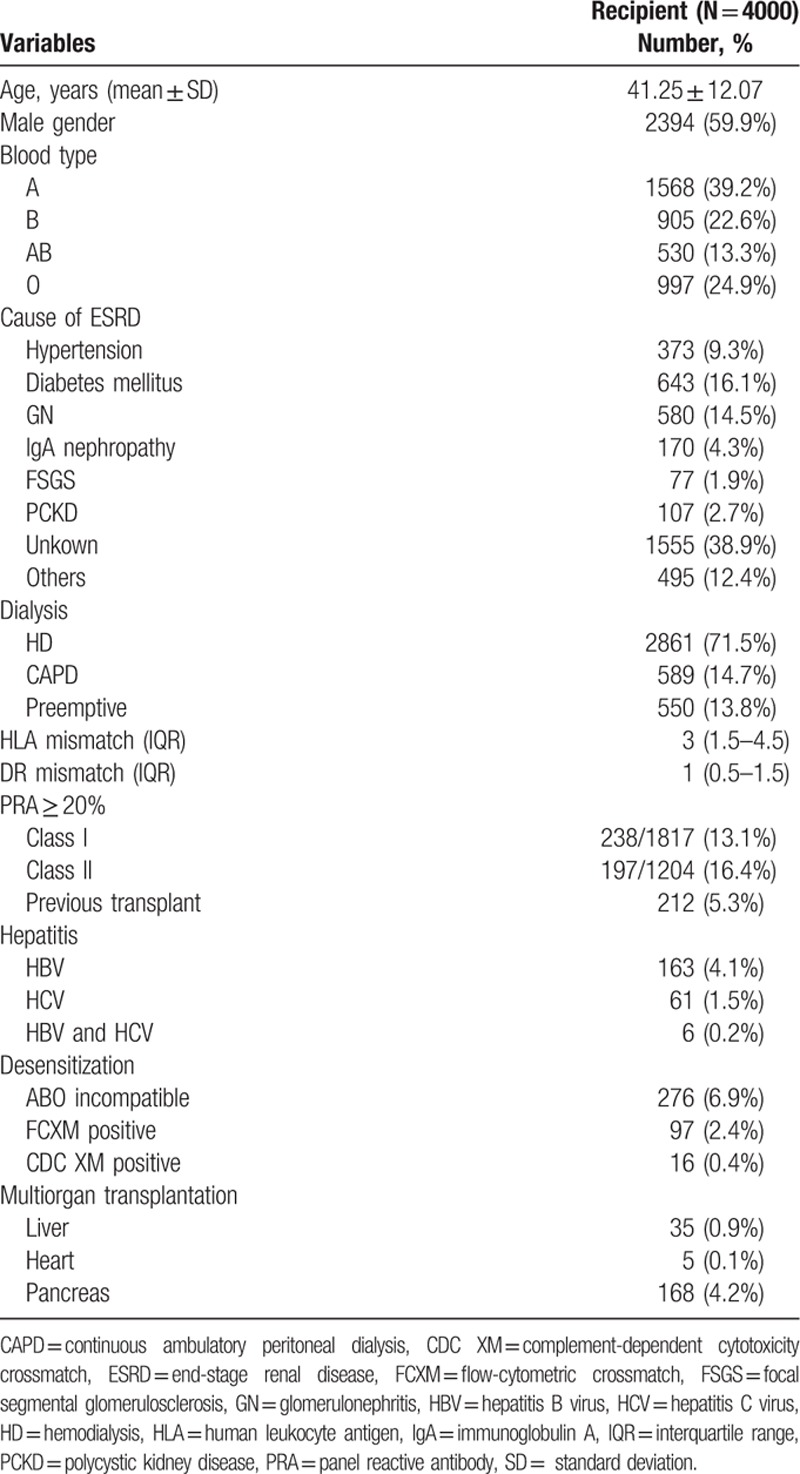
Recipient demographics and clinical characteristics.

**Table 2:**
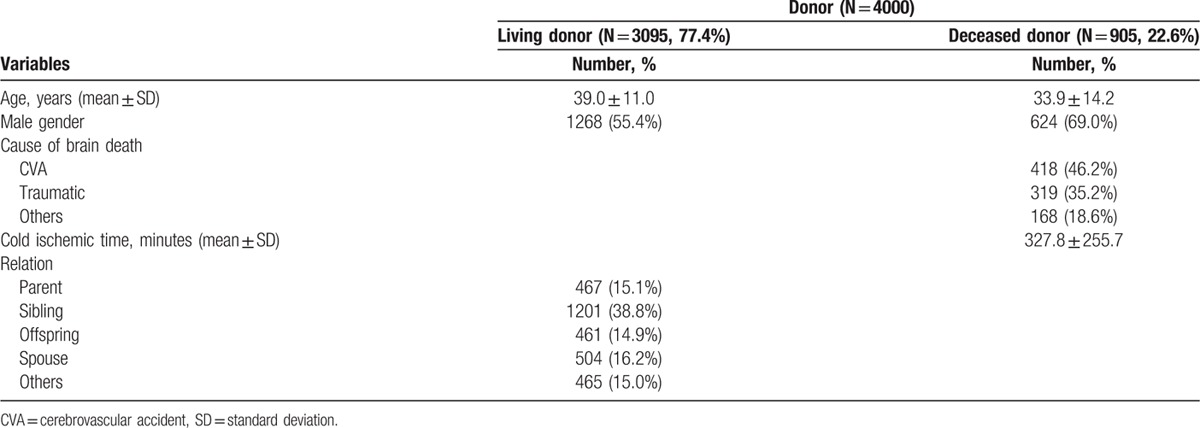
Donor demographics and clinical characteristics.

### 3.2 Long-term PS and GS after kidney transplantation

**Figure 2:**
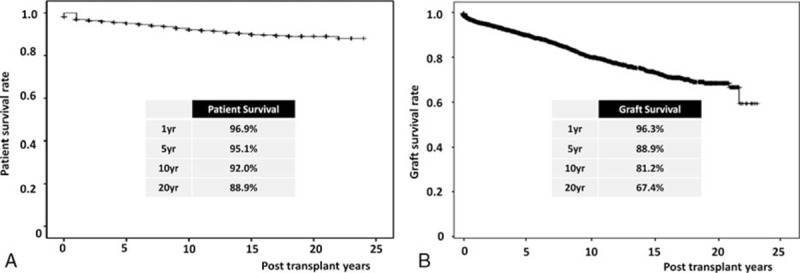
Long-term patient and graft survival after kidney transplantation. (A) Overall patient survival, (B) overall graft survival.

**Figure 3:**
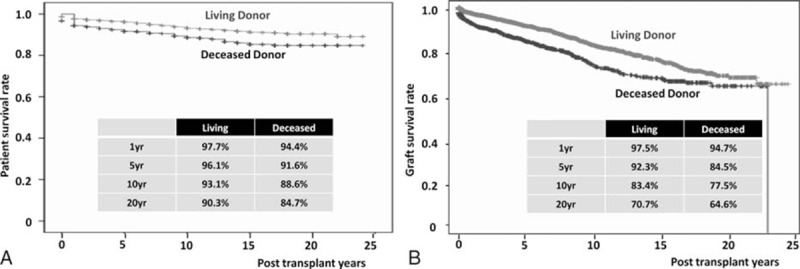
Long-term patient and graft survival after kidney transplantation stratified by living and deceased donors. (A) Overall patient survival, (B) overall graft survival.

**Figure 4:**
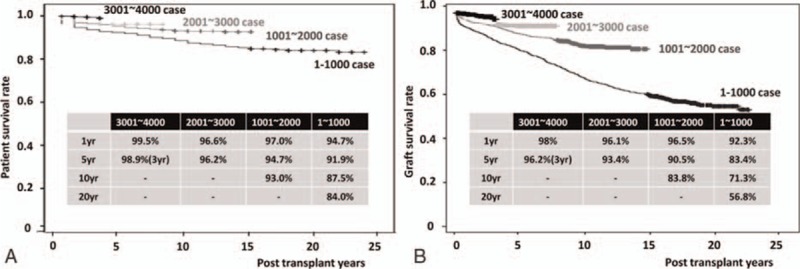
Long-term graft survival after kidney transplantation stratified by era. (A) Overall patient survival, (B) overall graft survival.

By the Kaplan–Meier analysis, the overall PS rates after KT at 1, 5, 10, and 20 years were 96.9%, 95.1%, 92.0%, and 88.9%, respectively (Fig. 2A). The overall GS rates after KT at 1, 5, 10, and 20 years were 96.3%, 88.9%, 81.2%, and 67.4%, respectively (Fig. 2B). The overall PS rates stratified by donor type at 1, 5, 10, and 20 years were 97.7%, 96.1%, 93.1%, and 90.3%, respectively, in living-donor kidney transplantations (LDKTs) and 94.4%, 91.6%, 88.6%, and 84.7% in DDKTs (Fig. 3A). The overall GS rates stratified by donor type at 1, 5, 10, and 20 years were 97.5%, 92.3%, 83.4%, and 70.7%, respectively, in LDKTs and 94.7%, 84.5%, 77.5%, and 64.6% in DDKTs (Fig. 3B). The overall PS rates stratified by era at 1, 5, 10, and 20 years were 94.7%, 91.9%, 87.5%, and 84.0%, respectively, in cases 1 to 1000. At 1, 5, and 10 years, they were 97.0%, 94.7%, and 93.0% in cases 1001 to 2000. At 1 and 5 years, they were 96.6% and 96.2% in cases 2001 to 3000. Finally, at 1 year, it was 99.5% in cases 3001 to 4000 (Fig. 4A). The overall GS rates stratified by era at 1, 5, 10, and 20 years were 92.3%, 83.4%, 71.3%, and 58.6%, respectively, in cases 1 to 1000. At 1, 5, and 10 years, they were 96.5%, 90.5%, and 83.8% in cases 1001 to 2000. At 1 and 5 years, they were 96.1% and 93.4% in cases 2001 to 3000. Finally, at 1 year, it was 98% in cases 3001 to 4000 (Fig. 4B).

### 3.3 Subgroup analysis

#### 3.3.1 ABO incompatible KT

**Figure 5:**
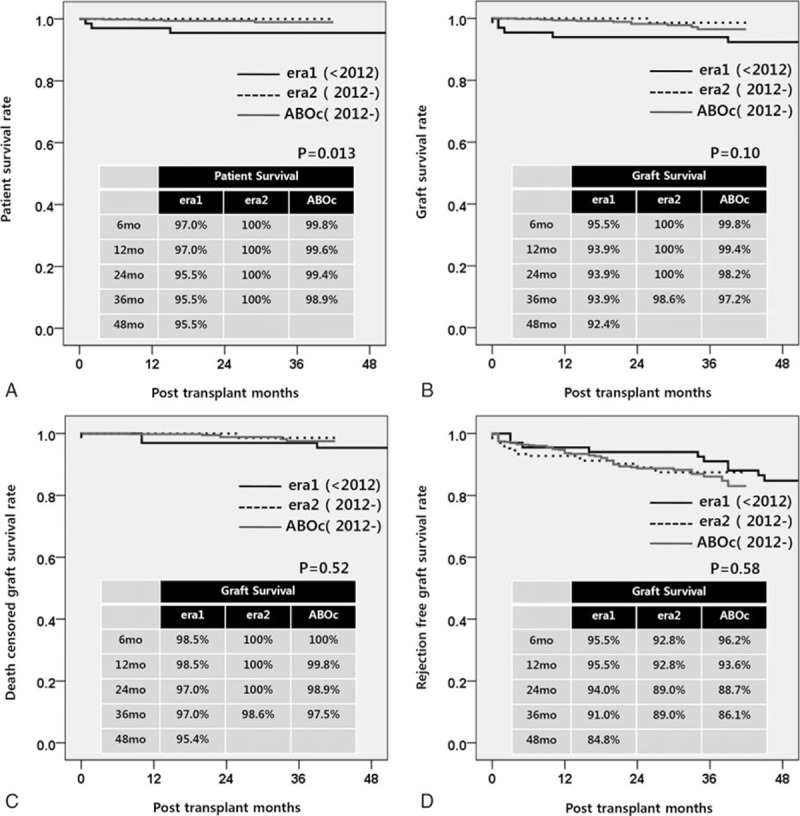
Long-term patient and graft survival in ABO-incompatible kidney transplantation stratified by era and ABO incompatibility. (A) Overall patient survival, (B) overall graft survival, (C) death censored graft survival, and (D) rejection free graft survival.

The subgroup cohorts for the subgroup analysis of ABOi KT comprised the control group of 600, the era 1 group of 67, and the era 2 group of 167. All patients underwent ABOi KT under the condition that the IgG and IgM Ab titers against blood groups A or B were below 1:8. The mean numbers of plasmapheresis before transplant were 4.3 ± 1.7 (SD) in era 1 and 3.6 ± 1.4 (SD) in era 2. The demographic and clinical characteristics of the patients are presented in Table 3. The patients in the era 1 group showed a significantly decreased PS in comparison with the era 2 and control groups during the 3-year follow-up (era 1 vs era 2 vs control group; 95.5% vs.100.0% vs 98.9%, *P* = 0.013) (Fig. 5A). The overall GS rates among subgroups showed no significant difference during the 3-year follow-up (era 1 vs era 2 vs control group; 93.9% vs 98.6% vs 97.2%, *P* = 0.03) (Fig. 5B). There was also no significant difference in the 3-year death-censored GS rates (era 1 vs era 2 vs control group; 97.0% vs 98.6% vs 97.5%, *P* = 0.52) (Fig. 5C) or the 3-year rejection-free GS rates (era 1 vs era 2 vs control group; 91.0% vs 89.0% vs 86.1%, *P* = 0.58) (Fig. 5D). The patients in era 1 showed a higher rate of infectious complications, such as CMV infection and pneumonia, compared to the patients in the era 2 or control groups. Surgical complications, however, demonstrated no significant differences (Table 4).

**Table 3:**
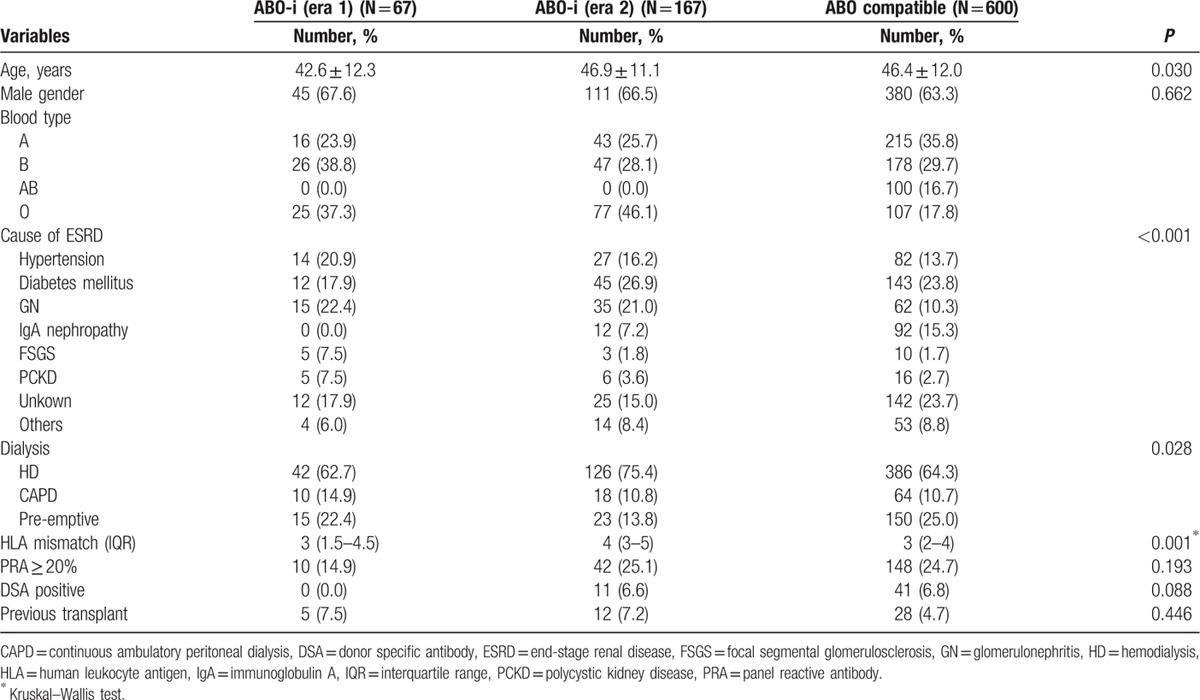
Demographics and clinical characteristics stratified by ABO incompatibility.

**Table 4:**
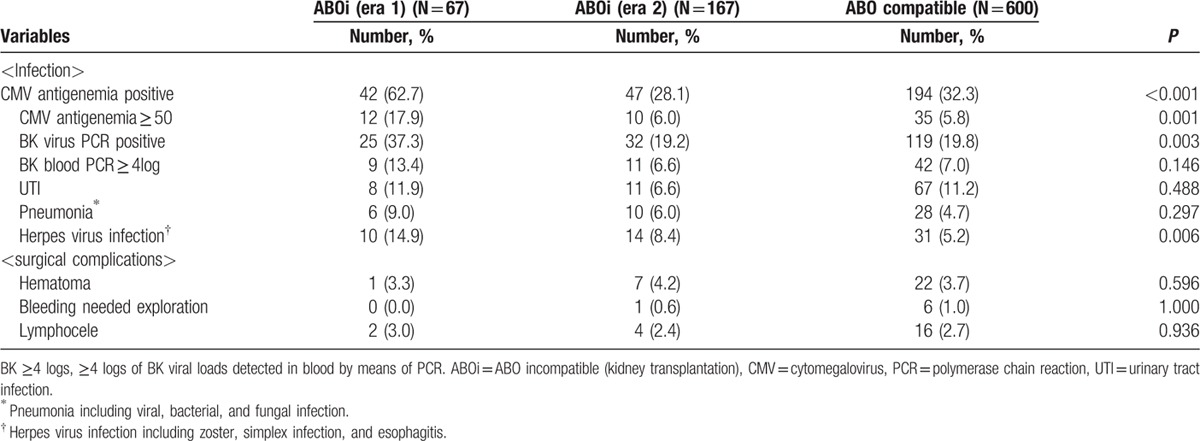
Infectious and surgical complications in ABO incompatible kidney transplantation.

#### 3.3.2 FCXM positive KT

After the patients were treated with a desensitization protocol, a total of 45 patients who had initially shown positive FCXM underwent KT. Among the 45 patients, 5 (11.1%) underwent KT in an FCXM-positive state. However, there was no acute rejection during the immediate postoperative period in these 5 patients. Only 1 patient experienced biopsy-proven rejection 7 months after transplantation. The mean number of plasmapheresis before transplant was 6.7 ± 4.2 (SD). The demographic and clinical characteristics of the patients are presented in Table 5.

**Table 5:**
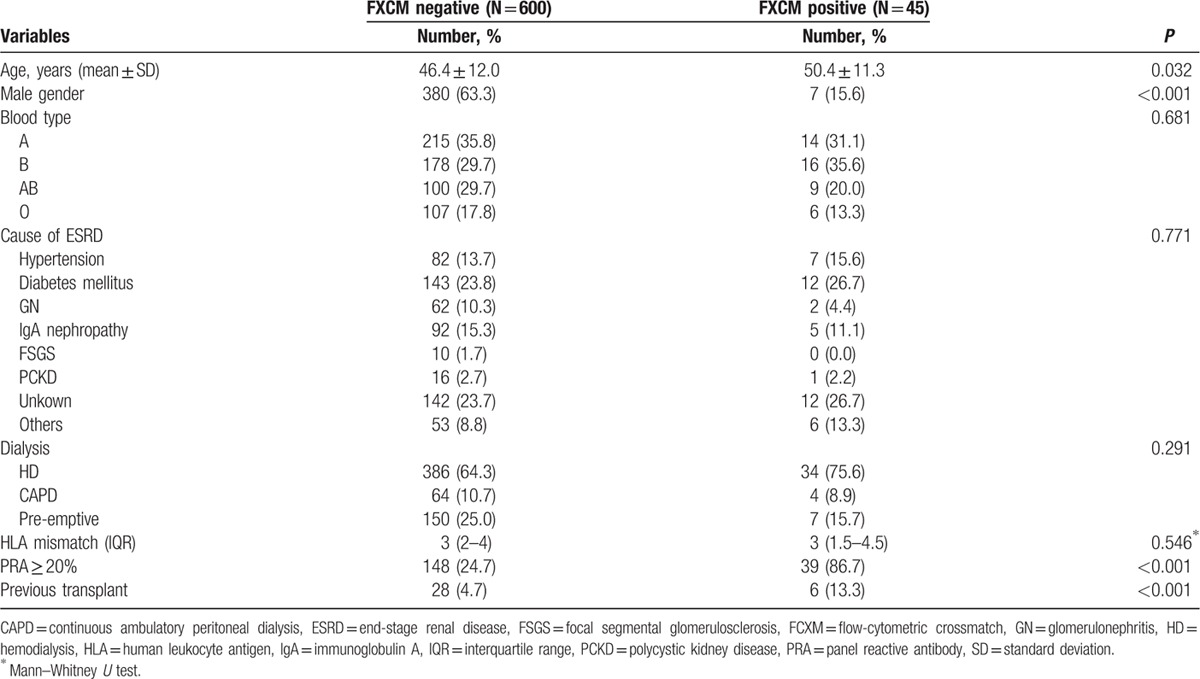
Demographics and clinical characteristics stratified by HLA incompatibility.

**Figure 6:**
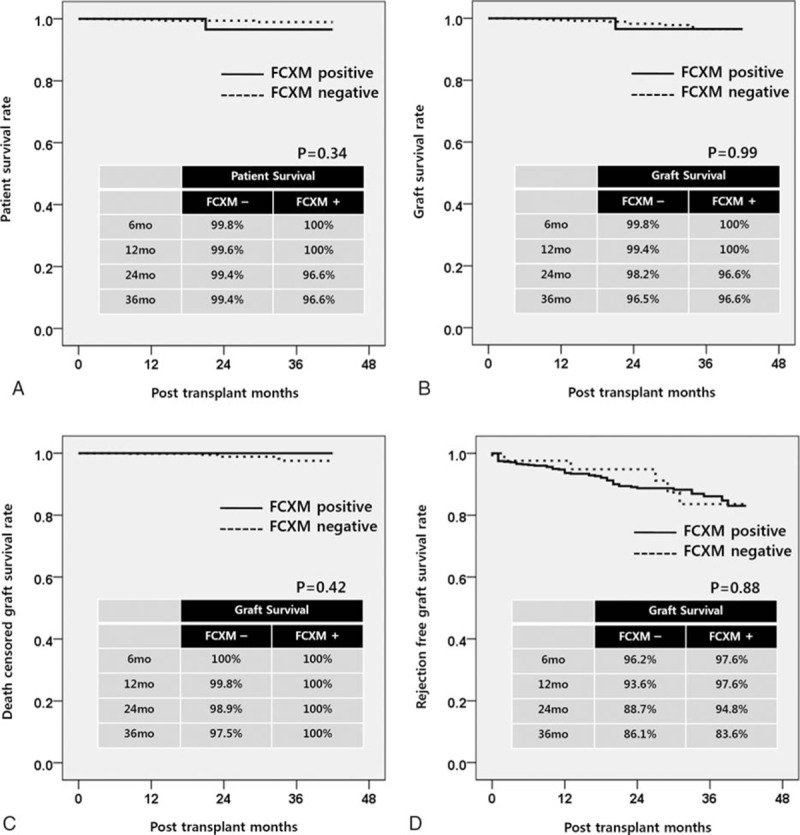
Long-term patient and graft survival in FXCM positive KT (A) overall patient survival, (B) overall graft survival, (C) death censored graft survival, and (D) rejection free graft survival.

The overall PS rate in patients who underwent FXCM postivie KT was not significantly different from that of the control group during the 3-year follow-up (*P* = 0.34) (Fig. 6A). The overall GS, death-censored GS, and rejection free GS also did not differ significantly between FXCM KT and control groups. (*P* = 0.99, 0.42, and 88)(Fig. 6B–D). The FXCM positive group showed a higher rate of surgical complications – including hematoma (3.7% vs 20.0%, *P* < 0.001), bleeding requiring operation (1.0% vs 6.7%, *P* = 0.002), and lymphocele (2.7% vs 8.9%, *P* = 0.020) – than the FXCM negative group. Infectious complications, however, demonstrated no significant differences (Table 6).

**Table 6:**
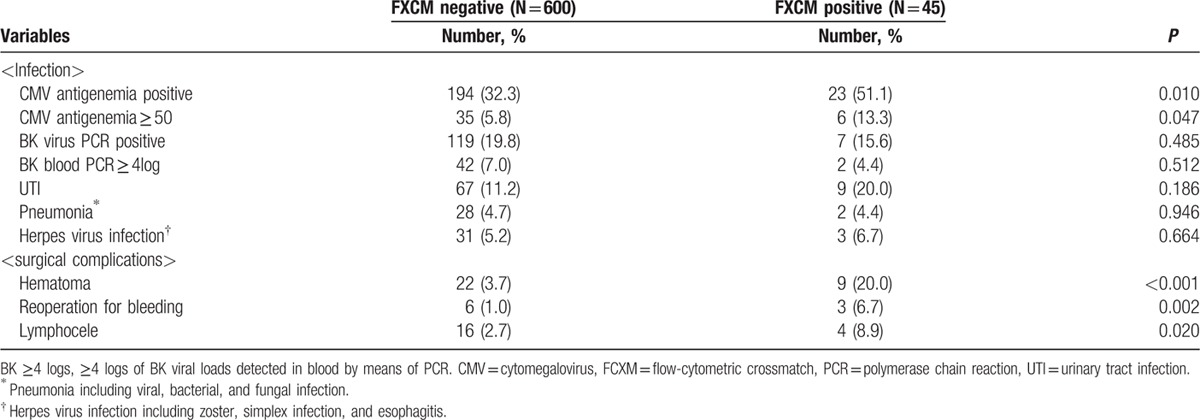
Infectious and surgical complications in FXCM positive kidney transplantation.

## 4 DISCUSSION

Here, we here report the patient characteristics, long-term PS, and GS, including ABO- and FCXM positive KT, for KT recipients at AMC. The annual number of KTs in our center increased during the study period. This increase resulted from the accumulation of experience in KT procedures and efforts to expand the criteria for acceptance as transplant or donor candidates. The recipient age ranged from 2 to 77 years old, and 5.8% of recipients were hepatitis B or C virus carriers. Immunological barriers (e.g., ABOi [6.9%], FCXM positive [2.4%], and positive CDC XM KT [0.4%]) are no longer contraindications. We have tried to overcome these barriers to improve the quality of life and survival of patients with ESRD.

The overall long-term death-censored GS rate in this study is comparable to that of other recent reports from large-scale studies. [^5^ ^6^ ^7^ ^8^] The outcomes for recipients have improved in the past 3 decades for several critical reasons. The development and proper use of new immunosuppressive agents was the main contributing factor. In the early days of KT, no induction agent was used, except in a few cases using Muromonab-DC3 (OKT3). After OKT3 was withdrawn from the market due to severe cytokine release syndrome, IL-2R-Ab (basiliximab) became the main induction agent at our center. Recently, antithymocyte globulin has been shown to be a good option for patients who need early steroid withdrawal or for immunologically high-risk groups.[ ^9^ ^10^] Cyclosporine as a maintenance immunosuppressant, which was revolutionary for organ transplantation, has been gradually replaced by tacrolimus. In our hospital, we still use cyclosporine, especially in patients who are vulnerable to infection or those who have suffered from side effects of tacrolimus, such as hyperglycemia, nephrotoxicity, and neuropsychiatric problems. Mycophenolate was the main antimetabolite used in our center, and mTOR inhibitors have been used for patients who exhibited malignancies and posttransplant lymphoproliferative disorder. ^[10]^

Living-donor recipients showed better long-term GS than deceased-donor recipients. In general, it is known that LDKTs have better GS rates than DDKTs. ^[6]^ The major disadvantage of deceased-donor transplants does not seem to be immunological disadvantages. Terasaki et al ^[11]^ showed that the kidney grafts from living unrelated donors with a higher degree of HLA mismatching than that of cadaveric grafts have high survival rates. This survival benefit in LDKT is possibly attributed to the fact that the kidney grafts in LDKT were uniformly healthy. ^[11]^ Najarian et al ^[12]^ also reported that the quality of early posttransplant function is a major predictor of long-term outcome in DDKT and that cadaveric recipients with immediate good function have outcomes similar to those of living-donor recipients.

A significant number of KTs are currently performed despite the barrier of HLA Ab or major ABO incompatibility. Although KTs are performed most safely in the absence of HLA or ABO barriers, the increasing organ shortage and waiting times have made KTs across HLA and ABO Ab barriers possible solutions. ^[13]^ Of the 4000 KTs undertaken at our hospital, 276 (6.9%) were ABOi KTs. The overall PS rate (era 1 = 95.5%, era 2 = 100.0%) at 3 years in our patients was similar to those of recent studies from Germany (95.6%), ^[5]^ the UK (91.9%), ^[14]^ and Japan (97%). ^[7]^ The death-censored GS rate (era 1 = 93.9%, era 2 = 98.6%) at 3 years in our KT recipients also showed no significant differences from the results in Germany (93.4%), ^[5]^ the UK (98.4%), ^[14]^ or Japan (93%). ^[7]^ Our subgroup analysis suggested that overall PS, GS, death-censored GS, and rejection free GS in ABOi KT showed no significant differences in comparison with ABO compatible KT if adequate immunosuppressive treatment was performed.

ABOi KT is now accepted as a good treatment option for patients with ESRD. Early postoperative infections, however, are major disadvantages. We also experienced lethal infectious complications, including 7 mortalities during era 1. Of these 7 patients, 5 died from infectious complications (2 cases of fungal pneumonia, 1 case of fungemia with pseudomembranous colitis, and 2 cases of urinary tract sepsis) within 2 months of transplantation. A UK group also reported early fatal infectious complications, mostly *Pneumocystis jiroveci* pneumonia. ^[14]^ Our center modified the desensitization protocol with a reduced dose of rituximab. Moreover, the level of tacrolimus was reduced from 8–10 to 3–8 ng/mL, the dose of MMF was reduced from 1.5 g a day to 1 g 1 week after transplantation, and cyclosporine replaced tacrolimus as the maintenance drug in patients 55 years or older. After modification of the desensitization protocol, infectious complications, including cytomegaloviremia (62.7% vs 28.1%, *P* < 0.001) and BK viremia (37.3% vs 6.0%, *P* = 0.001), decreased and PS improved significantly (*P* = 0.009) in era 2. If immunosuppression is modified according to host conditions, ABOi KTs can be performed safely with successful outcomes.

HLA incompatible KT in previously sensitized recipients has become increasingly popular because of the organ donor shortage in the past decade. ^[15]^ Montgomery et al ^[16]^ showed that LDKTs in patients with donor-specific HLA-Ab after desensitization provided a significant survival benefit as compared to waiting for a compatible organ. Our PS rate in patients with positive FCXM were 100.0% at 1 year and 96.6% at 3years, as compared with the rates of 92.0% and 79.9%, respectively, in the positive FCXM patients in the study of Montgomery et al. The overall PS, GS, death censored GS, and rejection free GS in FCXM positive KT showed no significant differences in comparison with the FCXM negative group. These results suggest that FCXM positive KT can be performed safely with successful clinical outcomes. However, adverse events especially bleeding complications, including hematoma (20.0%) and reoperation for bleeding (8.9%), showed a significant increase compared to the control group. Bleeding complications may have been associated with plasmapheresis, which depletes coagulation factors. ^[16]^ Kim et al ^[17]^ also reported that pretransplant blood urea level and number of plasmapheresis sessions were associated with an increased risk of bleeding after KT.

Our present study had several limitations of note. It was a retrospective study that covered a 25-year period and the immunosuppressive protocols, screening regimens for transplant candidates, dialysis techniques, and treatment regimens for infectious complications changed during that time. More stratified and detailed studies on immunosuppressive agents, immunological differences, surgical factors, recipient medical status before transplant, and donor graft function are needed.

In conclusion, the annual number of KTs increased, and the outcomes of KTs continually improved during the study period. ABO or HLA-incompatible KT can be performed safely with successful graft outcomea by modification of the immunosuppressive regimen according to the host conditions.
